# Analysis of PRICKLE1 in human cleft palate and mouse development demonstrates rare and common variants involved in human malformations

**DOI:** 10.1002/mgg3.53

**Published:** 2013-12-17

**Authors:** Tian Yang, Zhonglin Jia, Whitney Bryant-Pike, Anand Chandrasekhar, Jeffrey C Murray, Bernd Fritzsch, Alexander G Bassuk

**Affiliations:** 1 Department of Biology, University of IowaIowa City, Iowa, 52242; 2 Department of Pediatrics, University of IowaIowa City, Iowa, 52242; 3 State Key Laboratory of Oral Diseases, West China Hospital of Stomatology, Sichuan UniversityChengdu, China; 4 Department of Cleft Lip and Palate Surgery, West China Hospital of Stomatology, Sichuan UniversityChengdu, China; 5 Division of Biological Sciences, University of MissouriColumbia, Missouri, 65211

**Keywords:** Cleft palate, Prickle1, Shh, Vangl2

## Abstract

Palate development is shaped by multiple molecular signaling pathways, including the Wnt pathway. In mice and humans, mutations in both the canonical and noncanonical arms of the Wnt pathway manifest as cleft palate, one of the most common human birth defects. Like the palate, numerous studies also link different Wnt signaling perturbations to varying degrees of limb malformation; for example, shortened limbs form in mutations of *Ror2*,*Vangl2*^*looptail*^ and, in particular, *Wnt5a*. We recently showed the noncanonical Wnt/planar cell polarity (PCP) signaling molecule *Prickle1* (*Prickle like 1*) also stunts limb growth in mice. We now expanded these studies to the palate and show that *Prickle1* is also required for palate development, like *Wnt5a* and *Ror2*. Unlike in the limb, the *Vangl2looptail* mutation only aggravates palate defects caused by other mutations. We screened Filipino cleft palate patients and found *PRICKLE1* variants, both common and rare, at an elevated frequency. Our results reveal that in mice and humans PRICKLE1 directs palate morphogenesis; our results also uncouple Prickle1 function from Vangl2 function. Together, these findings suggest mouse and human palate development is guided by PCP-Prickle1 signaling that is probably not downstream of Vangl2.

## Introduction

Cleft palate is one of the most common congenital birth defects. Cleft lip/palate (CL/P) was reported at a rate of 75.9 per 100,000 births in 2003, with 3066 cases reported in the US alone (Martin et al. 2005). Although strong data link maternal smoking to cleft palate in offspring (Shi et al. 2008), a wide range of congenital insults and genetic errors can lead to cleft abnormalities, leaving the etiology of cleft palate mostly unknown (Stanier and Moore 2004; Gritli-Linde 2007; Gritli-Linde 2008; Dixon et al. 2011).

Mice have been used as genetic models to study the etiology of cleft palate. In mice, around embryonic day 10.5 (E10.5), the secondary palate arises from the internal side of maxillary processes, first growing vertically along the side of the tongue (E12.5–E13.5) and then, around E14, growing upward and horizontally, populating the region above the tongue. At E15, the medial-edge epithelia of the two shelves fuse to render the continuous, intact palate (Gritli-Linde 2007; Bush and Jiang 2012). Disturbances during any of these stages can lead to cleft palate (Ferguson 1988).

Recently, several studies implicated Wnt signaling disruption in the pathogenesis of human palatal malformations (Chiquet et al. 2008; Menezes et al. 2010; He and Chen 2012; Mostowska et al. 2012a,b2012b). For instance, single-nucleotide polymorphisms (SNPs) in *WNT5A* (wingless-type MMTV integration site family, member 5A) and *ROR2* (receptor tyrosine kinase-like orphan receptor 2) have been linked to cleft palate in humans (Chiquet et al. 2008; Wang et al. 2012). Supporting the correlation in humans, both *Wnt5a* and *Ror2* mutant mice have a complete cleft of the secondary palate (Schwabe et al. 2004; He et al. 2008). Clues to the underlying cause of cleft palate in *Wnt5a* and *Ror2* mutant mice were revealed in the altered cell proliferation, and overall defective cell migration (He et al. 2008), while, cell death and in vitro palate fusion were normal in these lines (He et al. 2008). These data suggest that the cleft palate phenotype is the secondary effect of a problem with palatal growth.

Although it has been shown that Wnt5a/Ror2 phosphorylates Vangl2 (VANGL planar cell polarity protein 2) and thus regulates planar cell polarity (PCP) pathway in limb development (Gao et al. 2011), the role of Vangl2 in palate development seems to be limited. VANGL2 mutations have not been associated with human cleft palate. Consistent with this, *Vangl2*^*lp/lp*^ or Vangl2^*−/−*^ mice do not have defects in palate closure (Kibar et al. 2001; Murdoch et al. 2001; Montcouquiol et al. 2003). In addition, *Vangl2*^*looptail*^ mutation does not increase the penetration of cleft palate in *Fzd2*^−/−^ (frizzled family receptor 2, Wnt receptor) embryos, *Fzd1*^*+/*−^ embryos, or *Fzd1*^*+/*−^; *Fzd2*^−/−^ embryos (Yu et al. 2010). However, *Vangl2*^*looptail*^ mutation slightly increases the penetrance of cleft palate in *Vangl2*^*lp/+*^; *Fzd2*^*+/*−^; *Fzd7*^−/−^ mice (Yu et al. 2012). These results not only suggest the redundancy of Fzd and Vangl in palate development, but also raise the question as to whether all components of the PCP pathway are essential in palate development.

To answer this question, we started out to analyze the function of PRICKLE1 (prickle homolog 1, OMIM: 608500) in human and mouse palate development. The mammalian Prickle1 is believed to be a core PCP protein: it is thought to be recruited by Vangl to the cell membrane and this protein complex is asymmetrically localized at one side of the cell, which is the foundation to establish cell polarity (Gubb et al. 1999; Barrow 2006; Kestler and Kühl 2008; Raz et al. 2008; Tao et al. 2009; McNeill and Woodgett 2010; Gao et al. 2011; Wang et al. 2011; Yang et al. 2013). Our previous work in the limb development has shown that Prickle1, like Vangl2, Ror2 and Wnt5a, is essential for limb development (Yang et al. 2013). We also proposed that Prickle1 might mediate part of the Wnt5a, Ror2, Vangl2 signaling in limb development. Therefore, we examined whether Prickle1 is essential for palate development like Ror2, or it is only downstream of Vangl2, thus lacking a palate phenotype on its own.

The human population we analyzed was the Filipinos, who have high rates of CL/P. It was reported that CL/P occurs in 1.94 per 1000 live births, and 47,969 newborns with CL/P over an 8-year period at one hospital in Philippine in 1997 (Murray et al. 1997). This study also found high recurrence rates in siblings of nonsyndromic cleft lip/plate (NSCLP) in 23 per 1000 live births, and of cleft palate only (CPO) in 14 per 1000 live births (Murray et al. 1997). Furthermore, epidemiological, genome-wide association (GWA) studies and candidate genes studies on Philippine population identified several critical causal genes and environmental exposure factors for CL/P (Vieira et al. 2005; Beaty et al. 2010; Dixon et al. 2011; Ludwig et al. 2012). We show that mice homozygous for the *Prickle1C251X* mutation have cleft palate similar to *Wnt5a* or *Ror2* mutants. This defect is associated with altered *Shh* expression. On the contrary, *Vangl2* mutation does not affect *Shh* expression. In addition, we found linked common, noncoding variants in *PRICKLE1* to cleft palate, and identified rare *PRICKLE1* variants in patients with cleft palate. We conclude that, in contrast to limb development, during palate development the function of Vangl2 is uncoupled from Prickle1 function.

## Material and Methods

### Human data

#### Samples

All patient DNA samples were collected with written informed consent following internal review board criteria, abiding by the Helsinki Treaty, and de-identified. We sequenced the seven exons of *PRICKLE1* in 87 nonsyndromic cleft lip and palate (NSCLP) individuals from Philippines. An additional 542 NSCLP individuals and 343 controls from Philippines were then similarly screened, to measure the frequency of the missense variants identified in the original screen (p.L380F [NM_153026.2:c.1138C>T] and p.R676W [NM_001144883.1:c.2026C>T]). Also, 221 large Filipino pedigrees (1032 nuclear families) with nonsyndromic cleft lip and cleft palate (NSCLP), cleft lip only (CLO), nonsyndromic cleft lip with or without cleft palate (NSCL/P) and cleft palate only (CPO) were genotyped for seven, tagging SNPs. Informed consent was obtained for all participants (University of Iowa approval numbers 199804081).

#### Sequencing

Primers were designed with Primer3 (http://biotools.umassmed.edu/bioapps/primer3_www.cgi) to cover all exons of *PRICKLE1*. Primers and PCR product details are in Table 1. PCR products were sequence by the Sanger method (Functional Biosciences, Inc., Madison, WI); and variants were identified using “Consed”. The Variant Effect Predictor (POLYPHEN2 and SIFT) from Ensemble database (http://www.ensembl.org/tools.html) was used to predict the functional effects of missense variants (McLaren et al. 2010).

**Table 1:** Primers of Prickle exons.

Exons	Primer name	Sequence	Annealing temperature
EXON1	F	GGTCGGGGGTAAGAGAAATG	60.0
	R	TGGTATTCCAGCATCTCAGTG	
EXON2	F	AGAGGCCAAACCCTGTACCT	60.0
	R	GGAGTTGGGGTTTATGAGCA	
EXON3	F	TTCCCTTTTTCTAGAGAGGCTGT	60.0
	R	TGCTAGTCCAGTCACCTACCC	
EXON4	F	AGGAAAGCCTGAGAATCCTG	60.0
	R	ATTTTGCTTGATGTAAACAGTGGA	
EXON5	F	TTTAAGAGCCAGTGTCTGTCCA	61.5
	R	CAAAGCTCATCAGCTGGAAC	
EXON6	AF	GCTCCCCCATACCCATAATC	60.0
	AR	TTCGAGAAAGGGTGTCATCA	
	BF	CAAGTTTCCTGGCCTCTCAG	60.0
	BR	CAGTCCATCTTGTGACTGTGC	
	CF	CCAGAGCCTTGCAAGTAAAAA	54.7
	CR	ACTGCGCCTGGCTTGAAT	
EXON7	AF	TTGAGATTGGAAATTTTCTTTGAA	54.7
	AR	TGCCGGATTTCAATGTCATA	
	BF	AACTGAGGGGTGGGAAGTGC	60.0
	BR	TCCAGAGAAAATCCTGCCTGA	
	CF	TGAATCGGTTTCTGGGACTC	60.0
	CR	ACATGGGCAAAGAAAGCACT	

#### Genotyping

Seven, tagging SNPs (rs12658, rs3747562, rs11181521, rs2406680, rs12309460, rs10880314, rs12581019) were genotyped using TaqMan SNP Genotyping Assays (Life Technologies, Grand Island, NY); and the results were analyzed with SDS 2.3 software (Applied Biosystems, Foster City, CA).

### Statistical analysis

FBAT (v1.73) (Horvath et al. 2001) was used to perform the TDT analysis. Odds ratios for each SNP were calculated from PLINK software (http://pngu.mgh.harvard.edu/purcell/plink/). To classify the types of variations, three criteria were set: very rare variants with an MAF less than 1%; rare variants with an MAF less than 5%; and common variants with an MAF above 5%. Significance levels adjusting for multiple comparisons using Bonferroni would be 0.05/28 (seven SNPs and four phenotypes).

### Mice

All animal treatment was approved by University of Iowa IACUC (ACURF 0804066) and (ADURF1109204). The *PrickleCys251X* mutant mice have been previously described (Tao et al. 2011; Yang et al. 2013). Noon, on the day of the vaginal plug visualization, was designated as embryonic 0.5 (E0.5). Embryos were fixed in 4% paraformaldehyde (PFA). Mice were genotyped as previously described (Yang et al. 2013).

The *Vangl2*^*Lp-m1Jus*^ mice carrying the D255E mutation were originally obtained from Dr. Olivier Pourquié (IGBMC, Illkirch, France). Mice were phenotyped and genotyped as described previously (Glasco et al. 2012).

### In situ hybridization

The probes for in situ hybridization were generated from the plasmid by in vitro transcription and then labeled with digoxigenin. *Shh*,*Bmp4*,*Prickle1*,*Fgf10*, and *Wnt5a* probes were previously described (Jones et al. 1991; Bitgood and McMahon 1995; Kraus et al. 2001; Pauley et al. 2003; Okuda et al. 2007; Glasco et al. 2012).

The embryos were hemi-sected in 0.4% PFA. For each probe, opposite halves from mutant and wild-type littermate embryos were labeled in the same tube, to minimize variability. The in situ hybridization protocol was previously described (Duncan et al. 2011). Whole-mount samples were digested with 20 μg/mL Proteinase K for 1 h. Each reaction was repeated at least once, at a given stage, until consistent results were achieved at least twice. Samples were imaged using a Leica M205 FA microscope with Leica Application Suite V3 (Wetzlar, Germany). All whole-mounted heads were imaged from the ventral side; and then images were compiled using CorelDRAW14.

The palate was sectioned coronally into 100 μm sections in 0.4% PFA using a Microtome. Anterior palate is defined as the region anterior to the molar tooth. Sections were digested with 20 μg/mL Proteinase K for 40 min and reacted for in situ hybridization (Duncan et al. 2011).

### Cryosection and H&E staining

Fixed mouse heads were incubated in 30% sucrose, 4% PFA, overnight, before sectioning. Samples were then frozen in OCT, in a tissue mold. Sections (20 μm) were cut in a cryostat at −22°C, and then transferred to room temperature on microscope slides. H&E staining was then performed on the samples using the OMRF H&E staining protocol (http://imaging.omrf.org/wp-content/uploads/2012/09/HandE_Protocol.pdf). Samples were mounted in Permount and imaged using Nikon E800 microscope (Tokyo, Japan).

### Proliferation test

Two hours before sacrifice, pregnant females were injected with PBS containing EdU (Invitrogen, Carlsbad, CA) at a concentration of 100 μg/gm body weight. Embryos were kept in 4% PFA, at least O/N, and sectioned with Microtome into 100 μm coronal sections. EdU Click-iT (Invitrogen) was performed on whole palate and corresponding coronal sections of wild-type and mutant palate, as per the instructions given by the manufacturer's manual. Sections were imaged using a Leica SP5 confocal microscope. For coronal sections, three optical levels at a 5 μm distance were counted for each section; the counts were averaged and the average value was counted as the number for that sample. For whole-mount palates, 50 μm image stacks were taken at 5 μm interval from the ventral surface.

### Quantification of palate length

First, a midline dividing the left and right head was defined. Objective markers were used to quantify the palate. Before the palate closure, the length of palate was measured from the junction between primary and secondary palate to the most posterior ruga which is labled with *Shh* expression. After palate closure (E14.5), The length of the palate was measured from the posterior tip of the *Shh* expression in primary palate to the most posterior dot of *Shh* expression in the palatal shelves. A line parallel to this defined midline was drawn and the length of the line was measured in Corel Draw ×4. Left and right palatal shelves were measures separately and the average of the palate lengths was used as the palate length for that sample.

## Results

### Rare *PRICKLE1* variants in human cleft palate

**Figure 1:**
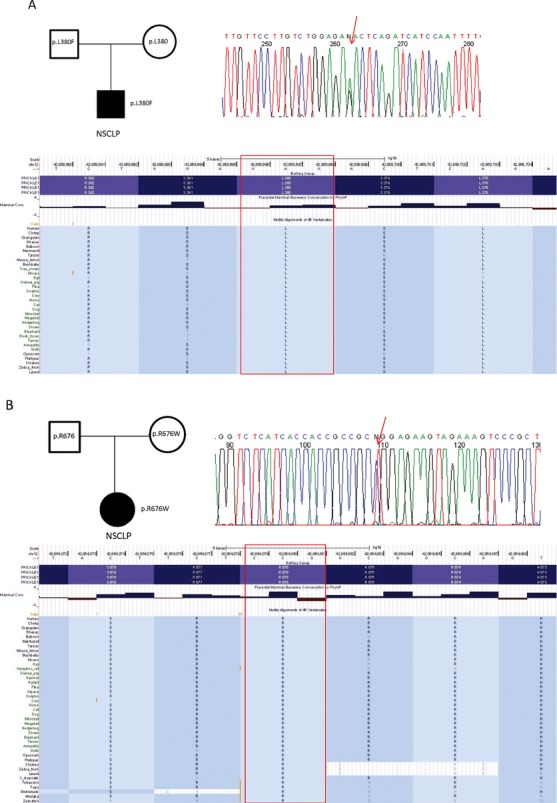
Pedigrees of affected families, representative chromographs, and evolutionary conservation of altered PRICKLE1 amino acids. Red arrows denote affected nucleotides in respective chromographs. The highly conserved amino acids altered in both families are indicated by a red box. The family in (A) has (NM_153026.2:c.1138C>T p.L380F) mutation. The mother and son have the mutation. The family in (B) has (NM_001144883.1:c.2026C>T p.R676W) mutation. The mother and son have the mutation.

To determine whether patients with nonsyndromic cleft lip and palate (NSCLP) could harbor variations in *PRICKLE1*, we first sequenced the entire coding region of *PRICKLE1* in a cohort of 87 NSCLP patients from the Philippines. From a total of 629 patients and 343 ethnically matched controls, variants were identified and analyzed. This screen detected two families (A and B) each harboring a rare, *PRICKLE1* variant that would be predicted to be deleterious—neither is represented in the 1000 Genomes Project, or the 8591 chromosomes from the National Heart Lung and Blood Institute (NHLBI) Exome Project; both were also absent in our Philippine controls (Table 2). Family A (Fig. 1A) harbors the p.L380F mutation (NM_153026.2:c.1138C>T), shared by the NSCLP-unaffected mother. *PRICKLE1* c.1138C>T alters an evolutionarily conserved residue PRICKLE1p.L380F (Fig. 1A) that lies possibly within a phosphorylation site (predicted by http://www.cbs.dtu.dk/services/NetPhos/ and Xue et al. 2008). Family B (Fig. 1B) harbors a p.R676W mutation (NM_001144883.1:c.2026C>T), also shared by the NSCLP-unaffected mother. *PRICKLE1*c.2026C>T alters a highly evolutionarily conserved residue p.R676W (Fig. 1B), predicted to be within conserved nuclear localization signals (Shimojo and Hersh 2006) (http://www.sbc.su.se/~maccallr/nucpred/ and http://nls-mapper.iab.keio.ac.jp). Phosphorylation and nuclear localization signals are necessary for Prickle1's localization to the nucleus, and thus these two mutations possibly affected PRICKLE1's normal function in the nucleus (Shimojo and Hersh 2003, 2006).

**Table 2:** The minor allele frequency of the variants.

Position (Hg19)	rs ID	Amino acid change	Alleles	NSCLP-Philippine	Control-Philippine	1000 Genome (CHB)	1000 Genome (JPT)	Polyphen2/SIFT
42866332			T/C	0.59%	–	–	–	
42863266	rs79087668	A124T	C/T	1.79%	–	10.80%	5.60%	Benign/Tolerated
42863262	rs34837068	V125A	A/G	11.90%	–	9.30%	5.60%	Benign/Tolerated
42859961	rs12230583		A/G	10.24%	–	23.70%	24.20%	
42858525		I437I	A/G	0.41%	0.61%	–	–	
42858698		L380F*	G/A	0.08%	0.00%	–	–	Benign/Deleterious
42858137	rs58707767		T/G	1.18%	–	4.10%	2.80%	
42854208	rs3747563	F633F	A/G	12.07%	–	30.40%	27.00%	
42854205	rs3747562	S634S	A/G	32.18%	–	45.40%	36.50%	
42853871	rs3827522	P746S	G/A	0.49%	0.46%	10.30%	5.60%	Benign/Tolerated
42854081		R676W*	G/A	0.08%	0.00%	–	–	Benign/Deleterious
42853997		Y704D*	A/C	0.08%	0.31%	–	–	Benign/Tolerated
42853541			C/T	0.61%	–	–	–	
42853520	rs1043652		G/A	10.37%	–	30.40%	27.00%	

* The missense mutation was absent in 1000 Genome and NHLBI ESP.

Although these two variants are rare, both were inherited from mothers without NSCLP, so they are not sufficient to cause NSCLP. If these variants contribute to NSCLP, then they do not show complete penetrance in either family. Such inheritance is consistent with previous reports of incomplete penetrance for NSCLP-associated genotypes, in both mice and humans (Juriloff 1982; Maestri et al. 1997; Parsons et al. 2008; Yu et al. 2010, 2012; Girardi et al. 2011; Nasser et al. 2012). The lack of any cleft palate abnormality in our previously described epilepsy patients with *PRICKLE1* mutations further demonstrates that not all *PRICKLE* variations will be associated with palate abnormalities (Bassuk et al. 2008; Tao et al. 2011).

In addition to finding rare *PRICKLE1* coding variants in the cleft palate cohorts, we evaluated the association between more common noncoding *PRICKLE1* SNPs and cleft palate. Transmission disequilibrium test (TDT) results demonstrated that rs12658 (3′UTR of *PRICKLE1*) is associated with cleft lip only (CLO, *P*-value 0.004, marginally above the multiple comparisons *P*-value of 0.002 for significance), and the C allele at rs12658 is protective for CLO with OR = 0.61 (Tables 2 and 3). Taken together, these data suggest that both common noncoding variants and rare coding variants in *PRICKLE1* may underlie palate malformations. To explore whether PRICKLE1 plays a role in causing NSCLP, we manipulated the Prickle1 gene in the mouse and then tracked palate development.

**Table 3:** TDT results by cleft group.

			CLO			NSCLP			NSCL/P			ALL		
rs ID	Position	Minor allele	afreq	fam#	P**	afreq	fam#	P**	afreq	fam#	P**	afreq	fam#	P**
rs12658^*^	42853084	C	0.45	71	0.0041	0.47	107	0.22	0.46	122	0.55	0.46	127	0.59
rs3747562	42854205	G	0.33	64	0.98	0.33	97	0.52	0.32	121	0.80	0.32	124	0.60
rs11181521	42882367	C	0.32	63	0.41	0.32	100	0.89	0.32	128	0.96	0.32	130	0.96
rs2406680	42908378	C	0.34	64	0.035	0.35	102	0.097	0.34	122	0.86	0.34	123	0.96
rs12309460	42933574	A	0.29	60	0.55	0.30	97	0.85	0.30	111	0.90	0.30	117	0.76
rs10880314	42940836	C	0.35	68	0.28	0.35	110	0.058	0.35	126	0.34	0.35	131	0.29
rs12581019	42962542	T	0.079	29	0.40	0.082	44	0.90	0.079	61	0.54	0.079	63	0.44

fam#, informative family number; # allele C is protective with OR = 0.61 from PLINK analysis;

** *P*, *P*-value; ALL, CLO&NSCLP&CPO&Unknown cleft type.

### *Prickle1*^*C251X/C251X*^ mice exhibit complete cleft secondary palate

To directly determine the role of Prickle1 in palate development, the palates of *Prickle1*^*C251X/C251X*^ mutant mice (which harbor a stop codon mutation at cysteine 251 of the mouse Prickle1 protein) (Yang et al. 2013) were examined during the course of development. This mutation form of protein lacks the third LIM domain and the c-terminal phosphorylation sites and nucleus translocation signals, which are essential for nuclear localization of Prickle1 protein (Shimojo and Hersh 2003, 2006; Mapp et al. 2011; Yang et al. 2013). Therefore, the mutation in the *Prickle1*^*C251X/C251X*^ mutant mice resembled the originally described mutation in human patients but, to an extreme extent.

**Figure 2:**
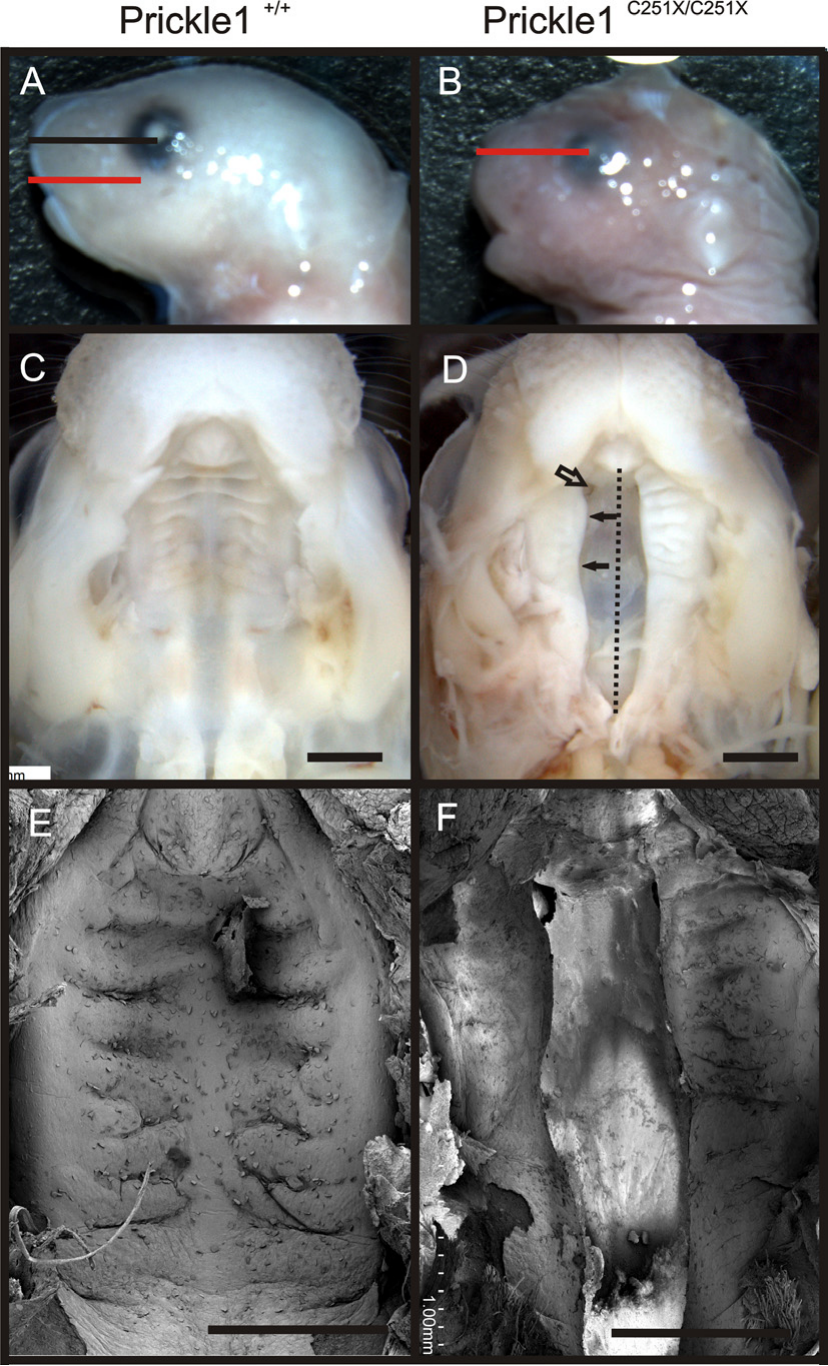
*Prickle1*^*C251X/C251X*^ mice have a shorter snout and complete secondary palate cleft at birth. (A and B) *Prickle1*^*C251X/C251X*^ mice have a shorter snout, defined by the distance from the snout tip to the center of the eyes. (C–F) Image of the palate from the ventral side after removal of the lower jaw of a fixed mouse. (D) The two palate shelves in the mutant do not contact or fuse; and one side is more affected (filled arrow). (E and F) SEM shows *Prickle1*^*C251X/C251X*^ mutants have cleft palate. Black line, the length of wild-type snout; black dotted line, the midline; red line, the length of the mutant snout; empty arrow, nostril; filled arrow, curved palate. Scale bar is 1 mm.

At E18.5, all *Prickle1*^*C251X/C251X*^ mutants had obvious shorter snout (Fig. 2A and B, compare black and red lines). We measured the length of the snout, which was defined from eye to tip of the snout, in a random selected subgroup of the embryos, and the mutants had ∼10% shorter snout (*n* = 6, *t*-test, *P* < 0.05). When we examined the palate, the secondary palate was completely open in *Prickle1*^*C251X/C251X*^ mice (Fig. 2C–F). In addition, the palate shelf of the mutant (arrow), often more deformed on the right palate shelf, was curved and farther from the midline (black dashed lines), compared to the left.

**Figure 3:**
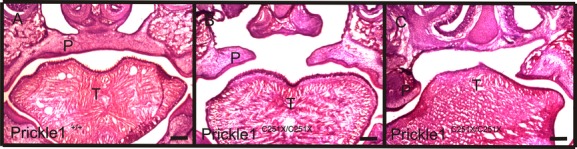
*Prickle1*^*C251X/C251X*^ palate elevation is affected in some mutants. A–C, The head from wild-type (A) and mutant (B and C) mice was coronally sectioned and stained with H&E. A: In wild-type mice, a uniform palate is formed. B, In the mutant, the two palate shelves rise to the horizontal position but do not fuse. C, In another mutant, two palate shelves fail to rise to the horizontal position. P, palate; T, tongue. Scale bar is 200 μm.

In *Wnt5a* mutants, the posterior palate shelves fail to rise to a horizontal position (He et al. 2008), so palate elevation was examined in E18.5 embryos of *Prickle1* mutant mice, in coronal sections of the palate. At this developmental stage, the wild-type palatal shelves had risen above the tongue and were fused at the midline (Fig. 3A). In the *Prickle1C251X* mutant, the palatal shelves had risen to horizontal but were too short to make the midline contact necessary for fusion (Fig. 3B). In some mutant mice, part of the palate shelves failed to rise normally (Fig. 3C).

### Palatal mesenchyme expresses *Prickle1* in an AP gradient

**Figure 4:**
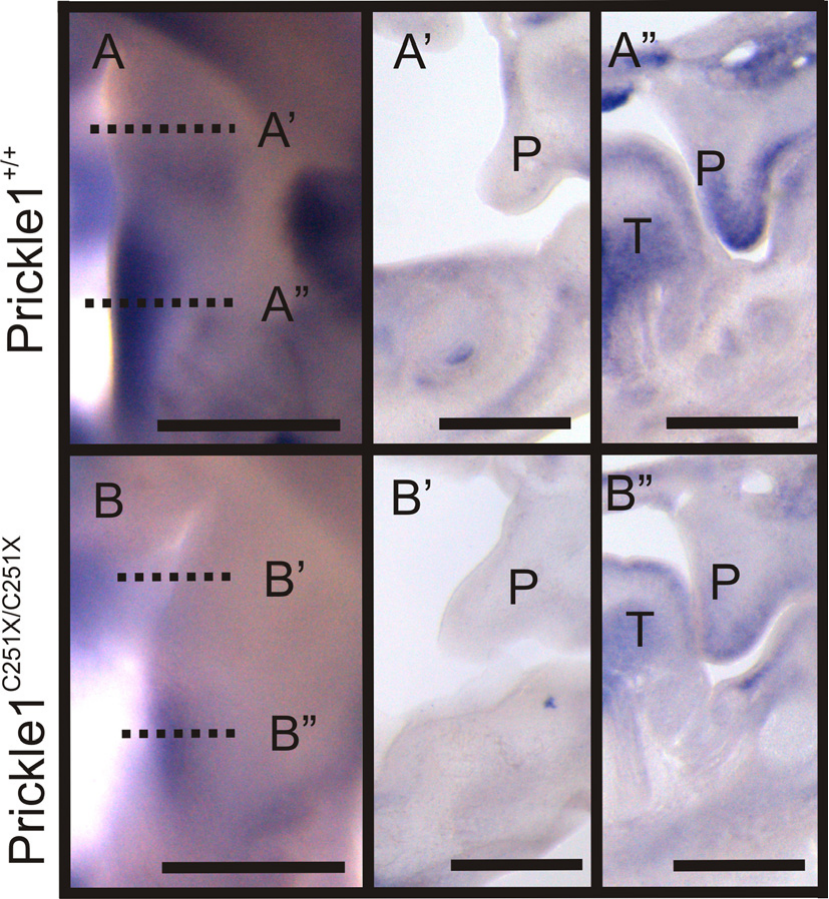
*Prickle1*mRNA is highly expressed in the mesenchymal cells of the posterior palate shelf, as shown by in situ hybridization. (A) Whole amount in situ hybridization shows Prickle1 is highly expressed at the posterior palate and weakly at the anterior palate. (A'–A”) Palate coronal sections from an E13.5 embryo show *Prickle1* expression was low in the anterior palate (p), but high in the mesenchymal cells in the posterior palate (p). (B–B”) *Prickle1* expression is downregulated in *Prickle1*^*C251X/C251X*^ mutants. T, tongue; P, palatal shelf. The scale bar is 500 μm.

To begin to uncover the mechanism by which *Prickle1C251X* mutation caused cleft palate, the pattern of *Prickle1* mRNA expression was visualized, by in situ mRNA hybridization of whole-mounted developing palates from E12.5 and E13.5 wild-type embryos. At E13.5, *Prickle1* expression was detected in a gradient pattern that was higher in the posterior and lower in the anterior palate (Fig. 4A). To refine the topological view, the palate was sectioned coronally and tested with in situ hybridization. Here, *Prickle1* expression was low in the anterior palate shelf, but was high in the mesenchyme of the posterior palate (Fig. 4A'–A”). The pattern of *Prickle1* expression at E12.5 was similar to that at E13.5 (data not shown). As the mutant form of the mRNA is not stable (Frischmeyer and Dietz 1999; Chang et al. 2007; Yang et al. 2013), *Prickle1* expression was expected to be lower in the mutants, as was the case in the developing limb (Yang et al. 2013). Consistent with this expectation, *Prickle1* expression was weak in *Prickle1*^*C251X/C251X*^ mice (Fig. 4B–B”), suggesting the reduced amount of Prickle1 protein in the mutant, if the protein is ever made.

### The *Prickle1C251X* mutation affects *Shh* expression

**Figure 5:**
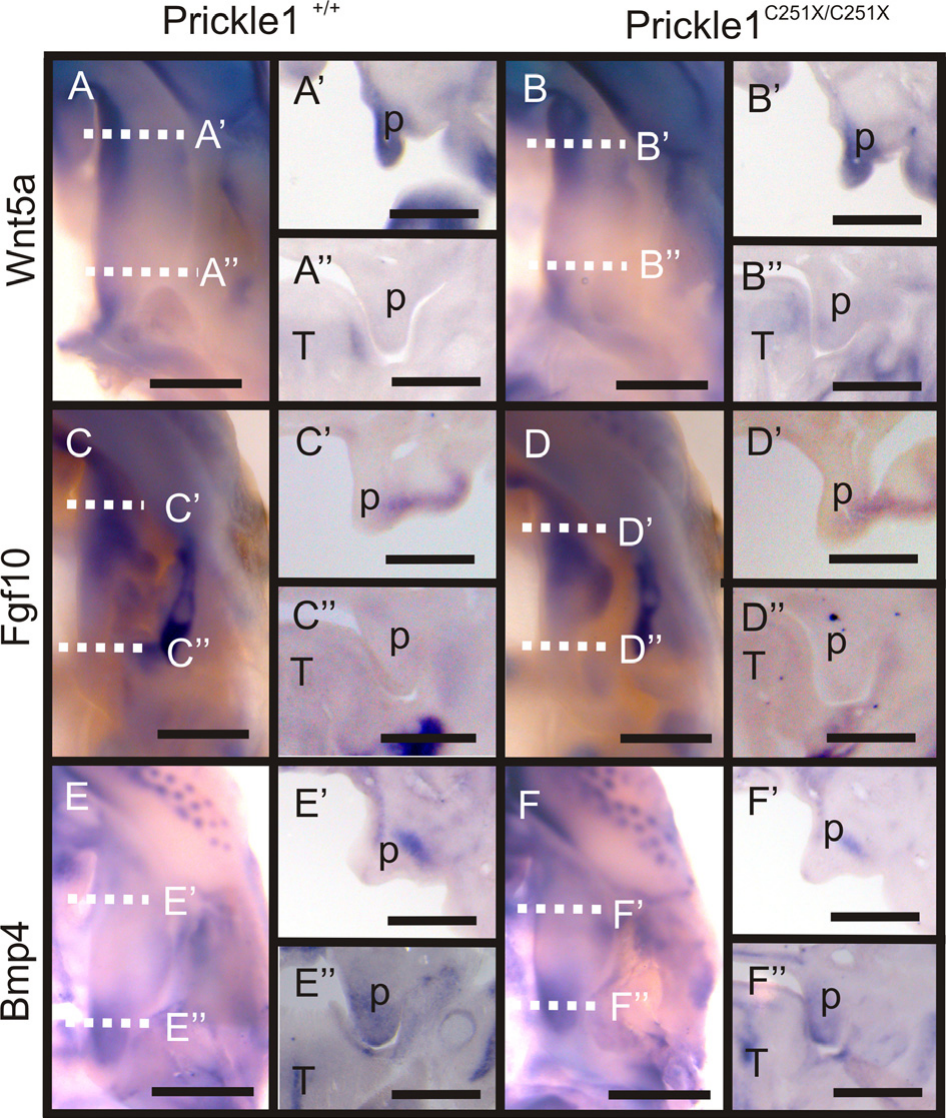
Expression pattern of several genes is not affected by the *Prickle1C251X* mutation shown by mRNA in situ hybridization on whole-mount palate and sections. (A–B”) *Wnt5a* is expressed in anterior palate mesenchyme and the posterior tip of the palate in both wild-type and the *Prickle1* mutant. (C–D”) *Fgf10* is expressed in the anterior palate mesenchyme in both wild-type and the *Prickle1* mutant. (E-F”) *Bmp4* is expressed in the posterior palate mesenchyme in both wild-type and the *Prickle1* mutant. T, tongue; p, palatal shelf. The scale bar is 500 μm.

Previously, we showed *Prickle1*^*C251X/C251X*^ mutant limbs are perturbed for the expression pattern of *Wnt5a* and *Bmp4*, genes known to be important for palate development (Zhang et al. 2002; He et al. 2008; Yang et al. 2013). In addition, *Wnt5a* mutant mice expressed *Bmp4* in an altered pattern but not *Fgf10* (He et al. 2008). Accordingly, *Prickle1*^*C251X/C25X*^ mice were assayed for the expression pattern of *Wnt5a*,*Bmp4* and *Fgf10*, but neither whole-mount nor coronal section in situ mRNA hybridization in the E13.5 mutant palate detected obvious change from wild-type palate (Fig. 5). Thus, the *Prickle1C251X* mutation did not affect the expression pattern of the three genes.

**Figure 6:**
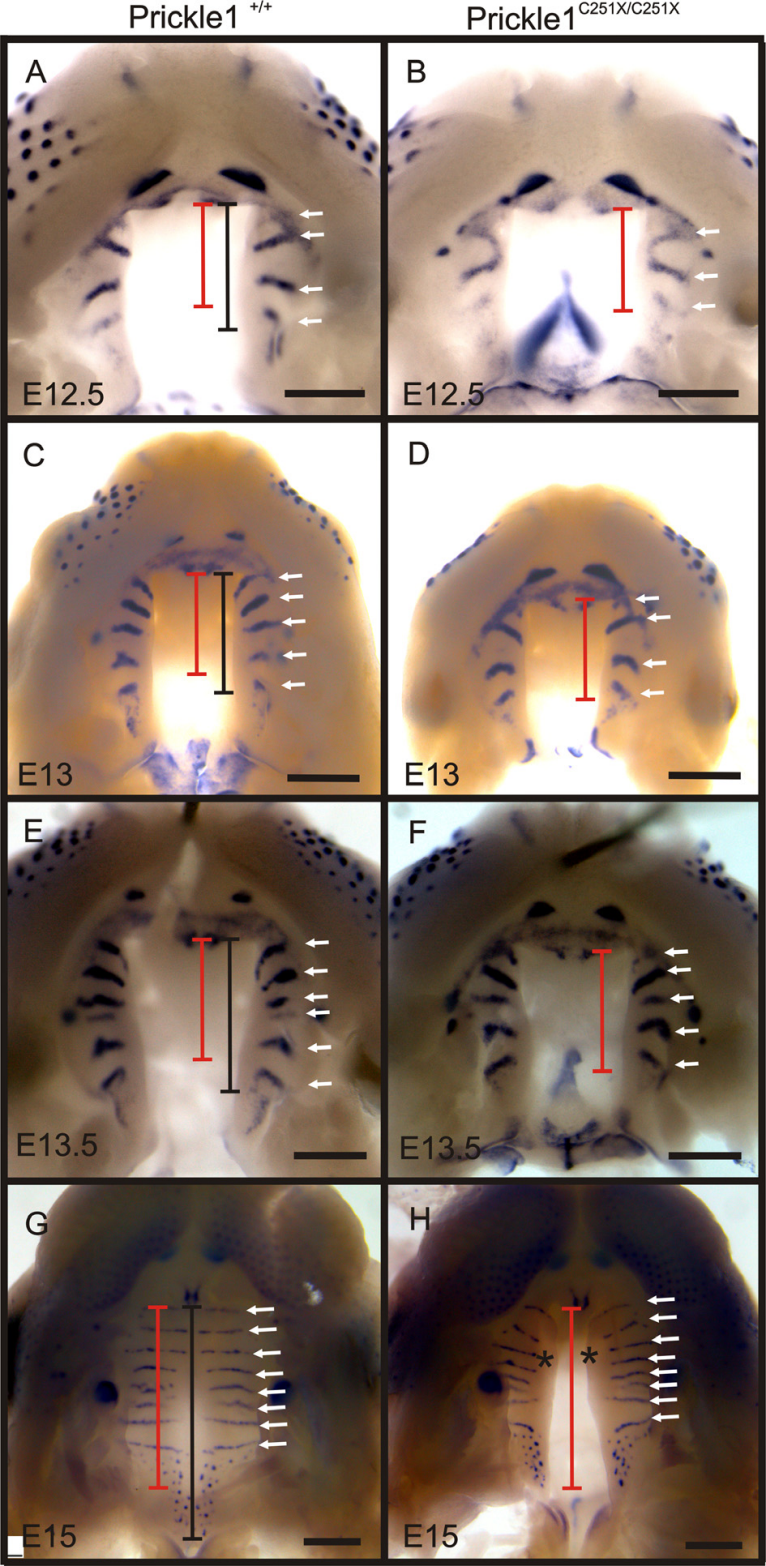
*Shh* expression is affected in *Prickle1C251X* mutants. (A–F) At E12.5, E13 and E13.5, *Shh* expression shows one ruga less in the mutant compared with wild-type palate (arrows), at each stage. (G and H) At E15, both wild-type palate and mutant have eight rugae but, in the mutant, the rugae in the anterior palate shelves are farther away from the medial edge (asterisks). Black bar, lengths of wild-type palate; red bar, length of the mutant palate; arrows, rugae; asterisks, medial edge of mutant palate. Images are taken from the ventral side. Anterior is up. Scale bar is 500 μm.

*Wnt5a*,*Bmp4* and *Fgf10* are expressed in the mesenchyme but not epithelium; so we then examined the expression of *Shh*, which is well-known to mediate the epithelial-mesenchyme interaction during palate development (Rice et al. 2004, 2006; Bush and Jiang 2012). *Shh* is expressed in the epithelium by the thickened rugae (Bitgood and McMahon 1995), which are transverse ridges on the secondary palate believed to aid in feeding and mastication (Bitgood and McMahon 1995). At E12.5, E13 and E13.5, *Shh* expression revealed the mutant palate developed fewer rugae compared to wild-type littermates (Fig. 6A–D, compare the number of arrows); however, by E15.5, both wild-type and mutant mice had developed the same number of rugae (Fig. 6E and F). Interestingly though, in E15 mutants, the medial edges of the anterior palatal shelves lacked *Shh* expression (Fig. 6G and H, asterisks), suggesting patterning abnormalities in the area. These results suggest that either rugae formation or *Shh* up-regulation was delayed in the mutant; however, this delay could be a consequence of the shorter palate shelves that had developed in the mutant.

The rugae provide objective markers to measure the length of the palate (Pantalacci et al. 2008; Welsh and O'Brien 2009), so we compared the length of palatal shelves in wild-type versus *Prickle1*^*C251X/C251X*^ mice (see materials and methods for defining the boundary). From E12.5 to E15, the palatal shelves (Fig. 6A–H, compare red and black bars) of the mutant were about 80.3 ± 2.83% of the wild-type palatal shelves (*n* = 8, *t*-test, *P* < 0.01).

**Figure 7:**
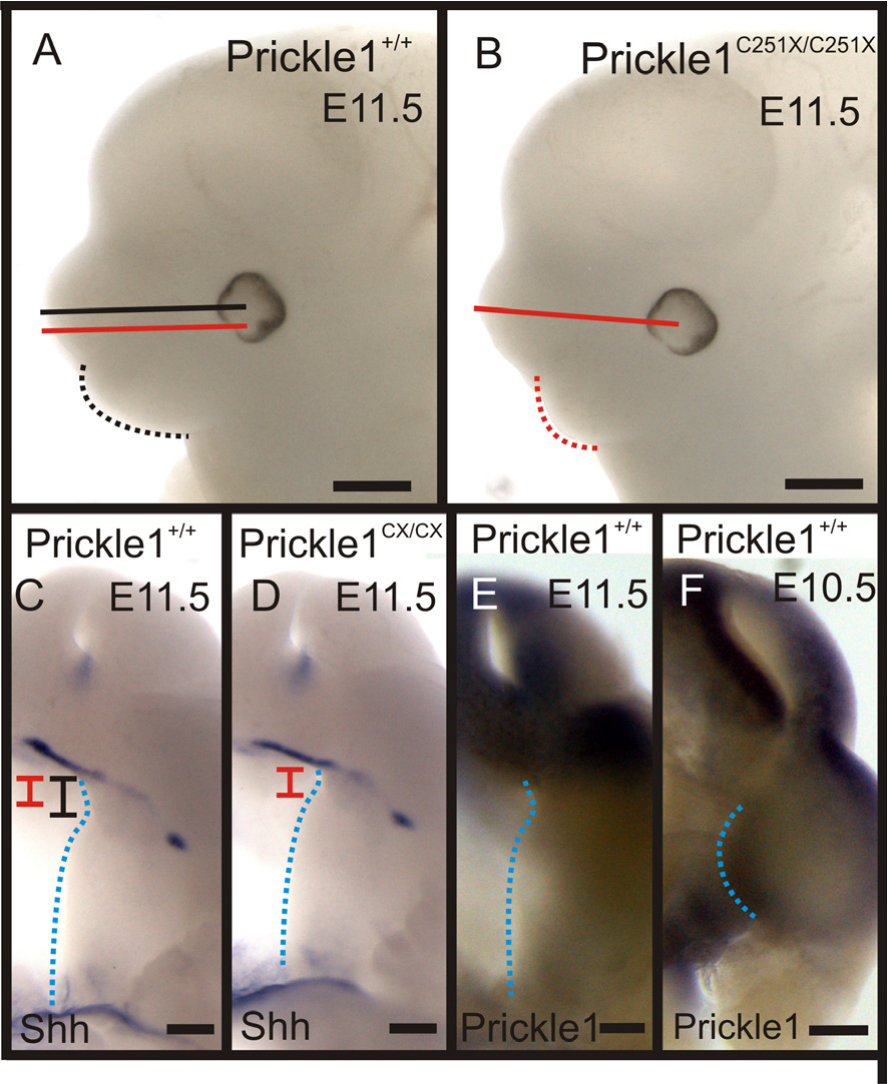
The mutant palate is shorter at E11.5. (A and B) Although the distance from the eye to the tip of the developing snout is the same in wild-type palate and mutant (compare the straight lines), the mutant maxilla processes are smaller (compare dashed lines). (C and D) *Shh*mRNA expression shows the developing anterior palate. At this stage, the Shh expression domain is already shorter in the mutant palate (compare the red and black bars, due to space limit, Prickle1^C251X/C51X^ is labeled as Prickle1^CX/CX^). (E) *Prickle1* is not expressed by the palate, but is highly expressed by the developing face/mouth. (F) *Prickle1*mRNA expression is detected internal maxilla processes at E10.5. Black bar, the length of wild-type anterior palate; black line, the length of the wild-type snout; blue dotted line, the medial boundary of the palate (or maxillary processes); red bar, the length of mutant anterior palate; red line, the length of the mutant snout. Scale bar is 500 μm in A and B, and 200 μm in C–F.

To understand whether the delay in *Shh* up-regulation in the mutant was a secondary effect from the shorter palate, the palate was examined at E11.5, a stage when the palate starts to develop (Gritli-Linde 2007). In the E11.5 mutants, the palates were shorter and curved differently than in wild-type littermates (Fig. 7A and B, dashed lines). *Shh* in situ hybridization offered insight into molecular changes at this stage: *Shh* was expressed weakly in the anterior palate; and, in the mutant, the *Shh*-positive region was already ∼30% shorter than that of the wild-type palate (Fig. 7C and D, black and red bars). *Shh* was also only weakly expressed at the palate posterior tip (Fig. 7C and D). Unexpectedly, although the palates in Prickle1^C251X/C251X^ embryos were already shorter, at this stage, we did not detect *Prickle1* expression in the palate (Fig. 7E, dotted blue line). However, there was strong *Prickle1* expression in the external maxillary processes that will develop into the cheeks and mouth (Fig. 7E). It is possible that Prickle1 is expressed weakly at this stage in the palate, below the detection threshold of whole-mount in situ hybridization. However, the shorter palate in the mutants at this stage suggests that Prickle1 has an earlier effect on palate development. Therefore, we examined *Prickle1* expression 1 day earlier before the initiation of palate development at E10.5 (Fig. 7F). At this stage, *Prickle1* was expressed by the internal maxillary processes (Fig. 7F, blue dotted line), which will extend medially into palatal shelves around E11.5. These results together suggest *Prickle1* mutants have shorter palatal shelves due to shorter maxilla processes at the onset of the palate development.

### Proliferation rate is reduced in the posterior of Prickle1^C251X/C251X^ palate

**Figure 8:**
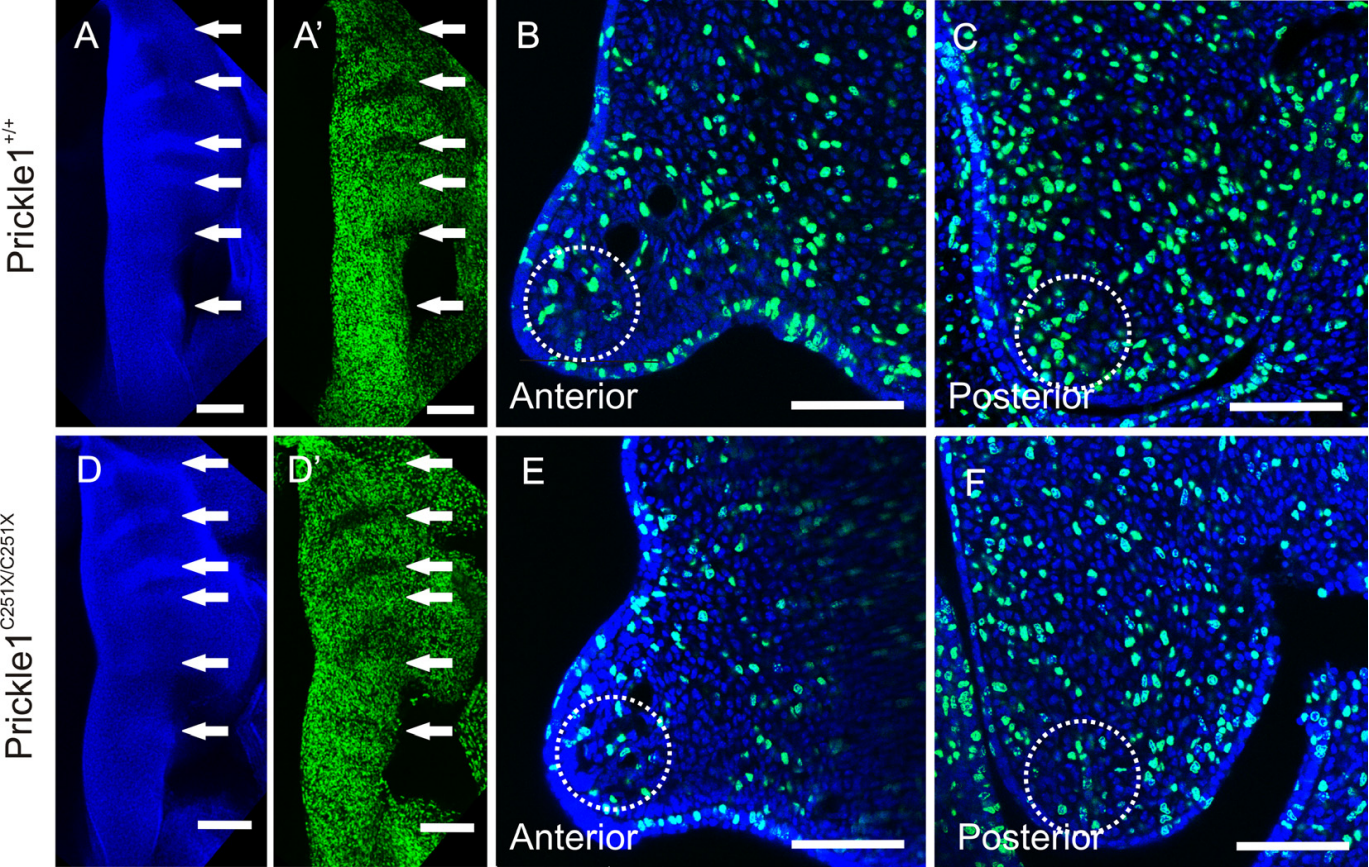
Proliferation is reduced in the posterior palate. (A and D) Hoechst staining showing the nuclei of the ventral side palate. Rugae contain more cells. (A' and D') EdU staining shows most cells in the rugae are not proliferating. (B and E) The coronal section of the anterior palate shows no obvious difference in proliferation. (C and F) There are fewer cells proliferating in the coronal section of the posterior palate in the mutant. Arrows, rugae; circles, the region where proliferating cells/total cells are quantified. The scale bar is 100 μm in B and C and E and F, and 200 μm in the rest.

The rugae are the organizational center of palate development. Cells in the rugae do not proliferate as fast as those cells between the rugae (Pantalacci et al. 2008; Welsh and O'Brien 2009). It is possible that this pattern in cell proliferation might be upset by delayed upregulation of *Shh*. To test this, cell proliferation was tracked with EdU in whole palate shelves. The images taken from the ventral side of E13.5 animals (Fig. 8A–A' and D–D') show that cells were more less densely packed in the gap between the rugae (Fig. 8A and D, arrows); nevertheless, the densely packed cells comprising the rugae were mostly EdU-negative (Fig. 8A' and D', arrows). The wild-type and mutant mice showed no clear difference in the proliferation pattern. When proliferation was measured in coronal sections (Fig. 8B and C and E and F), fewer cells in the posterior palate were proliferating (Table 4). And as the posterior cells will migrate to the anterior (He et al. 2008), if fewer cells grow in the posterior palate, then ultimately, fewer will be available to migrate to populate the anterior palate. This could be the cause of the smaller palate in mutant mice.

**Table 4:** Proliferation rate (proliferating cells/100 cells) in the palate.

	Anterior (*n* = 4)	Posterior (*n* = 4)
Prickle1^+/+^	19.5 ± 3.1	27.0 ± 5.8
Prickle1^C251X/C251X^	19.2 ± 2.7	20.2 ± 2.1*

* *P* < 0.05.

In the developing limbs of *Prickle1* mutants, we previously found that apoptosis changed more rapidly than in wild-type mutants (Yang et al. 2013). However, TUNEL test for apoptotic cells did not show any obvious changes in the apoptosis in the palate (data not shown).

### Vangl2lp mutation does not affect *Shh* expression

**Figure 9:**
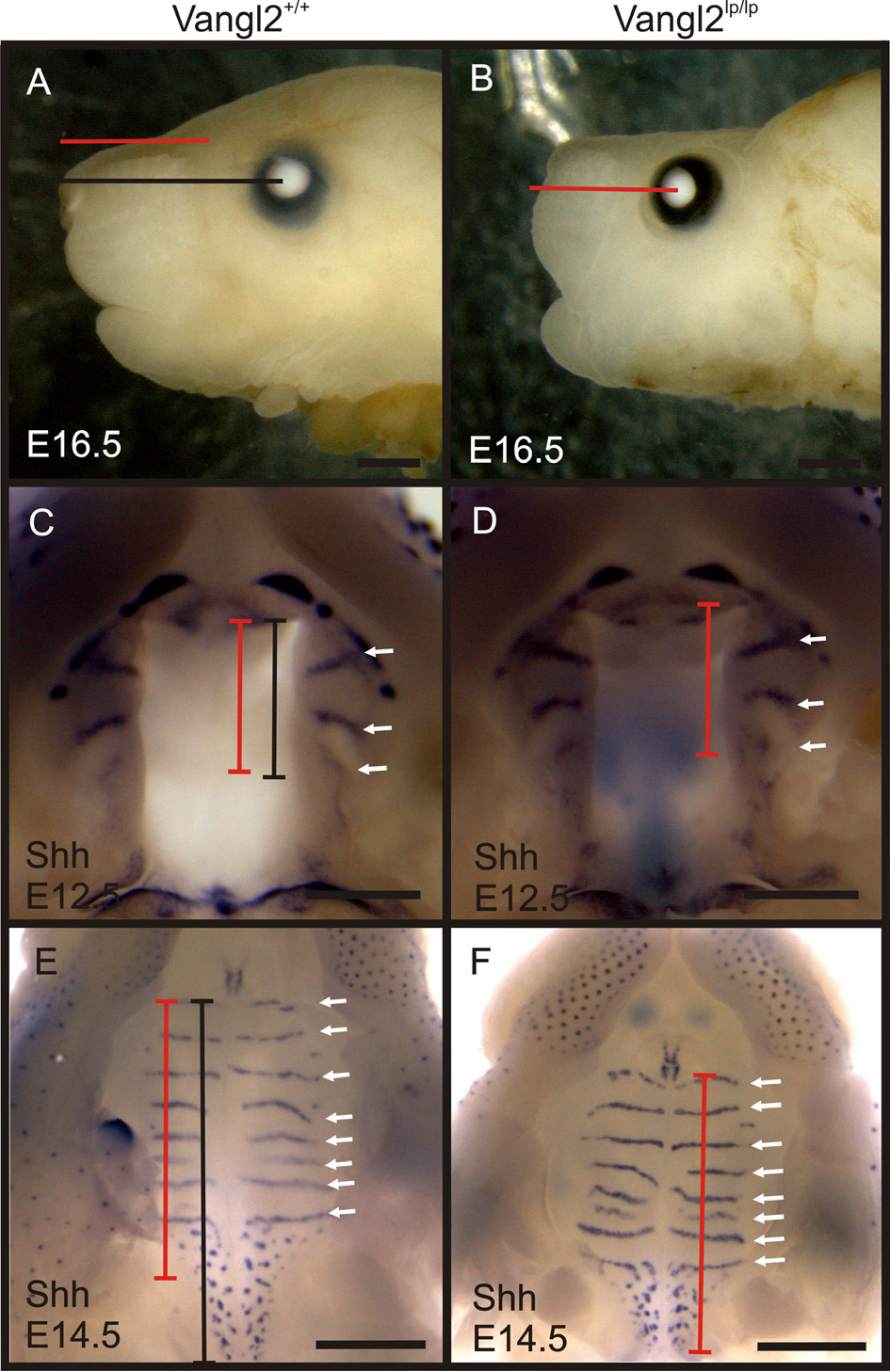
*Vangl2lp* mutation does not cause cleft palate. (A and B) At E16.5, the snout is shorter in the *Vangl2*^*lp/lp*^ mutant (compare the black and red lines). (C and D) At E12.5, both wild-type and mutant palates have developed three rugae, as visualized by *Shh* expression. (E and F) At E14.5, an intact palate has formed in both wild-type and *Vangl2lp/lp* mice. In addition, *Shh*mRNA expression shows the relatively normal rugae development, although the mutation palate is shorter than the wild-type littermate. Arrows, rugae; black bar, length of the wild-type palate; black line, length of the wild-type snout; red bar, length of the mutant palate; red line, length of the mutant snout. The scale bar is 500 μm.

Given that *Vangl2*^*lp/lp*^ mutants have shorter snouts similar to *Prickle1*^*C251X/C251X*^ (Fig. 9A and B), we asked whether the palate AP growth was affected similarly and whether *Shh* expression was affected. *Shh* in situ hybridization was used to visualize the palate patterning (Fig. 9C–F). Interestingly, the mutant and wild-type palatal shelves were of almost equal length at E12.5, but by E14.5 the mutant shelves were shorter than those of the wild-type shelves. More importantly, the *Shh* expression pattern showed rugae formation was not affected at either E12.5 or E14.5 (Fig. 9C–F, white arrows). These results together suggest that a shorter snout alone does not cause cleft palate and that *Vangl2* mutation does not affect *Shh* expression. This normal *Shh* expression in *Vangl2looptail* mutants also supports that abnormal *Shh* expression might be the cause of smaller palate and thus cleft palate in *Prickle1C251X* mutants.

## Discussion

### *PRICKLE1* in human cleft palate

Mutations in noncanonical Wnt signaling genes, such as *WNT5A* and *ROR2*, have been linked to human cleft palate (Chiquet et al. 2008; Wang et al. 2012) but this is the first study to examine human pedigrees for *PRICKLE1* mutations associated with cleft palate. We identified two rare *PRICKLE1* variants in individuals with NSCLP that are absent in controls. In addition, there is supportive evidence for an association of common variants in *PRICKLE1* with NSCLP although this also does not reach genome-wide significance (Ludwig et al. 2012). This association in human cleft palate cases is supported by the strong phenotype in *Prickle1*^*C251X/C251X*^ mice.

### Possible interaction between Wnt5a/Ror2 and Prickle1

The limbs of *Wnt5a, Ror2*,*Vangl2* and *Prickle1* mutant mice are shorter, to various extents, than those of wild-type counterparts (Yamaguchi et al. 1999; Raz et al. 2008; Gao et al. 2011; Wang et al. 2011; Yang et al. 2013), supporting the widespread idea that these four genes are part of the mammalian PCP pathway (Gubb et al. 1999; Barrow 2006; Kestler and Kühl 2008; Tao et al. 2009; McNeill and Woodgett 2010). Thus, we hypothesized that *Prickle1*-deficient mice would develop similar palatal defects as *Wnt5a* or *Ror2* mutants. Consistent with our hypothesis (and Wnt5a as well as Ror2 data [Schwabe et al. 2004; He et al. 2008]), the *Prickle1* mutation caused a completely cleaved palate (Fig. 1).

In mice, the posterior tip and anterior region of the palate express *Wnt5a* (Fig. 4), while *Ror2* and *Prickle1* are expressed in an opposite gradient along the palate (Fig. 3) (He et al. 2008). The overlapping expression of the three genes is consistent with the hypothesis that they are in the same signaling pathway. However, unlike the downregulation of *Wnt5a* expression in *Prickle1*^*C251X/C251X*^ mutant limbs (Yang et al. 2013), *Wnt5a* expression did not appear to change in the mutant palate at E13.5. Therefore, if Wnt5a, Ror2 and Prickle1 interact in the palate, then they likely do so via a different mechanism than the one they use to interact in the limb.

*Vangl2lp* mutants do not develop cleft palate but had shorter snout. In addition, mice homozygous for mutant *Vangl1* are viable and fertile; and *Vangl1* knockout mice were not reported to have cleft palate (Torban et al. 2008). The clear involvement of Prickle1 but not of Vangl1/2 in palate development suggests that Prickle1 functions independently of Vangl1/2. Moreover, these results demonstrate that Prickle1 does not always interact with Vangl1/2 to mediate Wnt/PCP signaling.

Combined, our data suggest that, in the palate, Prickle1 might mediate the Wnt5a/Ror2 signal. How and why Vangl1/2 is uncoupled from this signal cascade in the palate remains unclear.

### Effect of Prickle1 on palate development

At E10.5 before the development of palate, the internal maxillary processes express *Prickle1*. At E11.5, even though *Prickle1* mRNA expression is not detected by whole mount in situ hybridization, the anterior palate of the mutant is already shorter than the wild-type palate. These results suggest *Prickle1C251X* mutation affects palate development partially by disrupted maxillary development. The shortened palate of mutant is further affected by delayed *Shh* upregulation or rugae formation, which is the signaling center of palate development (Welsh and O'Brien 2009).

*Prickle1* is highly expressed by the posterior palate at E12.5 and E13.5. Supporting the role of Prickle1 in the posterior palate, we show proliferation is reduced in the posterior palate but not in the anterior palate. How does defective proliferation in the posterior palate lead to complete cleft of the whole secondary palate? One possible explanation is defective cell migration. It was shown that posterior palatal mesenchyme migrates anteriorly and anterior mesenchyme migrate medially (He et al. 2008). This migration requires Wnt5a/Ror2 signaling (He et al. 2008). If Prickle1 is part of this signaling, then the posterior mesenchyme probably cannot migrate anteriorly and the anterior mesenchyme cannot migrate medially. This leads to defects along AP axis and medial-lateral axis.

### *Shh* expression is affected in the *Prickle1C251X* mutation palate

At E11.5, before the first ruga forms, we found that the *Prickle1* mutant palate was already shorter than the wild-type palate. In addition, the *Shh* expression region is also affected by *Prickle1C251X* mutation.

Rugae are secondary signaling centers that coordinate the elongation of the palatal shelves (Welsh and O'Brien 2009). The rugae determine the expression pattern of several genes of the same signaling network (e.g., *Notch1*,*Fgf9* and *p63* [Welsh and O'Brien 2009]). In our *Prickle* mutant palates, although *Bmp4*,*Fgf10*, and *Wnt5a* mRNA expression was unchanged, *Shh* expression was delayed, consistent with a slower overall growth of the mutant palate. As *Shh* upregulation and rugae formation require separation by a minimal distance (Pantalacci et al. 2008), the delayed rugae formation might be secondary to the short mutant palates. It is also possible that delayed rugae formation further delayed the formation of new signaling centers, which further impaired palate development. More data are needed to further reveal how these molecular interactions operate. Nevertheless, at later stages (after the wild-type palate has fused at the midline; E15), the mutant, unfused palate forms the same number of rugae as the wild–type palate, demonstrating that rugae development is not disrupted, only delayed. However, we noticed that in the anterior palate of the mutant, the medial edges of the palate shelves do not develop rugae, suggesting that the patterning is perturbed on medial edge of the palate.

### Differential growth effect of Prickle1 and Vangl2 on palate and snout AP growth

Although both Prickle1 and Vangl2 affect palate and snout AP extension, they have different effects. *Prickle1*^*C251X/C251X*^ mutants have shorter palatal shelves from E11.5 to E15, which suggests *Prickle1C251X* mutation has an early effect and a late effect on palate development: it causes smaller maxillary processes, which in turn leads to shorter palate; the shortened palate affects *Shh* expression, which further affects palate development. On the contrary, *Vangl2lp* mutation does not affect palate development until E12.5. After E12.5, *Vangl2lp* mutation starts to affect palate AP extension.

On the contrary, *Vangl2lp* mutation has similar effect on the AP extension of the snout to the *Prickle1C251X* mutation. The shortened snout but no palate closure defects in the palate of *Vangl2*^*lp/lp*^ mice compared with *Prickle1*^*C251X/C251X*^ mice suggest shorter snout is not directly responsible for cleft palate.

In conclusion, we have shown that *PRICKLE1* variants are associated with cleft palate in humans and a dysfunctional Prickle1 in mice causes a completely cleaved palate. This growth defect is associated with smaller maxillary processes, delayed rugae formation, and reduced proliferation in the posterior palate. However, Vangl2, classic partner of Prickle1 in the limb, is responsible for snout growth as Prickle1, but does not also cause cleft palate nor change the expression of *Shh*, which reveals that the function of Prickle1 can be uncoupled from Vangl1/2. Prickle1 adds to the recently discovered complexity of gene expression regulation in facial development (Attanasio et al. 2013) by showing a surprising flexibility in the use of what is usually considered consistent aspects of its signaling pathway.
